# The prognostic value of TPM1–4 in hepatocellular carcinoma

**DOI:** 10.1002/cam4.4453

**Published:** 2021-11-30

**Authors:** Zhihui Tian, Jian Zhao, Yusheng Wang

**Affiliations:** ^1^ Gastroenterology Ward One Shanxi Province Cancer Hospital Taiyuan Shanxi China

**Keywords:** bioinformatics analysis, hepatocellular carcinoma, prognosis, risk model, tropomyosins

## Abstract

**Background:**

Despite advances in multiple disciplinary diagnoses and treatments, the prognosis of hepatocellular carcinoma (HCC) remains poor. Some evidence has identified that the aberrant expression of tropomyosins (TPMs) is involved with some cancers development. However, prognostic values of TPMs in HCC have not been thoroughly investigated.

**Methods:**

Original TPM1–4 mRNA expression of TCGA HCC data and GTEx was downloaded from UCSC XENA. Oncomine database and GSE46408 were used for verification. Clinical stages and survival analysis of TPM1–4 in HCC were performed by GEPIA2. cBioPortal was utilized to assess TPM1–4 gene alteration in HCC. TIMER2.0 was used for investigating the relevance of TPM1–4 to tumor‐infiltrating immune cells in HCC. Additionally, we constructed a TPM1–4 prognostic model to explore the value of TPM1–4 for prognostic evaluation in HCC. LinkedOmics was applied to elucidate TPM3 co‐expression networks in HCC.

**Results:**

This present study showed that TPM1–4 was upregulated in all HCC tissues, and TPM3 overexpression was correlated with poor survival outcomes in patients with HCC. Besides, TPM3 amplification was the main altered type in TPM1–4 genetic alteration, which affected the prognosis of HCC patients. The risk model revealed that TPM1, TPM2, and TPM3 were applied to risk assessment of HCC prognosis, among which TPM3 expression was significantly higher in the high‐risk group than that in the low‐risk group. Univariate and multivariate cox regression analyses indicated that TPM3 may be an independent prognostic factor of HCC prognosis. In addition, TPM3 co‐expression genes mainly participated in the cell cycle by maintaining microtubule cytoskeleton in HCC progression. TPM1–4 was associated with some tumor‐infiltrating immune cells in HCC.

**Conclusion:**

Our study detected that the expression level of TPM1–4 was all remarkably elevated in HCC, suggesting that TPM1–4 may serve an important role in HCC development. High TPM3 expression was found to be correlated with poor overall survival, and TPM3 may be an independent prognostic factor for HCC.

## Graphical abstract

Our study detected the expression of TPM1–4 was all significantly upregulated in hepatocellular carcinoma (HCC), suggesting TPM1–4 may serve as an important role in HCC development. High TPM3 expression was found to be associated with poor overall survival, and TPM3 may be independent prognostic factor for HCC.

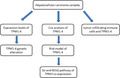

## 1 INTRODUCTION

Primary liver cancer is the sixth most frequent malignancy and the third leading cause of cancer‐related death worldwide, second only to lung cancer and colorectal cancer.^1^ Hepatocellular carcinoma (HCC) is the dominant histologic type of liver cancer and accounts for around 75%–85% of the total liver cancer cases.^2^ It is estimated that there were approximately 900,000 million new cases of primary liver cancer and 800,000 deaths from primary liver cancer globally in 2020 according to GLOBOCAN 2020 database.^1^ Statistically, the incidence of liver cancer in China accounts for about 55% of all worldwide cases, which is still a serious threat to public health.^2^ Despite advances in multiple disciplinary diagnosis and treatments, the prognosis of HCC patients remains poor due to the high possibility of recurrence and metastasis.^3^, ^4^ Thus, further exploration for underlying molecular mechanism may provide new promising diagnostic and therapeutic strategies for HCC.

As first described in 1946,^5^ tropomyosin (TPM) is a two‐chained α‐helical coiled‐coil actin‐binding protein that is widely expressed in muscle and nonmuscle cells.^6^ Apart from stabilizing the cell skeleton,^7^ TPM has been also confirmed to participate in some physiologic processes like cytoplasmic division, cell motility, cell apoptosis, and signal transduction.^8^ At present, four TPM genes have been identified in mammals, named TPM1, TPM2, TPM3, and TPM4.^9^ Previous studies show that TPM mutation in muscle cells is associated with a variety of muscle diseases including familial hypertrophic cardiomyopathy,^10^ myasthenia gravis,^11^ and arteriosclerosis obliterans.^12^ In addition, abnormal expression of TPM has been reported to involve with tumorigenesis and tumor progression.^13^ TPM1 is regarded as a tumor suppressor and overexpression of TPM1 can induce cancer cell apoptosis in the progression of cancer.^14^ TPM2 may be a biomarker candidate for some cancers. Several studies demonstrated that TPM2 is downregulated in human esophageal squamous cell carcinoma^15^ and colorectal cancer,^16^ whereas it is upregulated in ovarian cancer^17^ and breast cancer.^18^ The upregulation of TPM3 was reported to participate in the development of liver cancer.^19^ Moreover, the TPM3 gene can fuse with ALK and NTRK1 gene through translocation to induce the occurrence of inflammatory myofibroblastoma^20^ and papillary thyroid carcinoma.^17^ These results suggest that TPM3 is a pro‐tumor factor in some cancers. Like the role of TPM2 in cancers, upregulated TPM4 expression is found to promote hepatic carcinogenesis^21^ and suppress tumorigenesis of colon cancer.^22^

Previous studies have identified the key role of TPM1–4 in some cancers, especially the role of TPM3 and TPM4 in HCC development. However, prognostic values of TPM1–4 in HCC have not been thoroughly investigated. In this study, we aim to systematically analyze the correlation of TPM1–4 with clinic pathologic features and prognosis in patients with HCC.

## 2 METHODS

### 2.1 Original data sources

**FIGURE 1:**
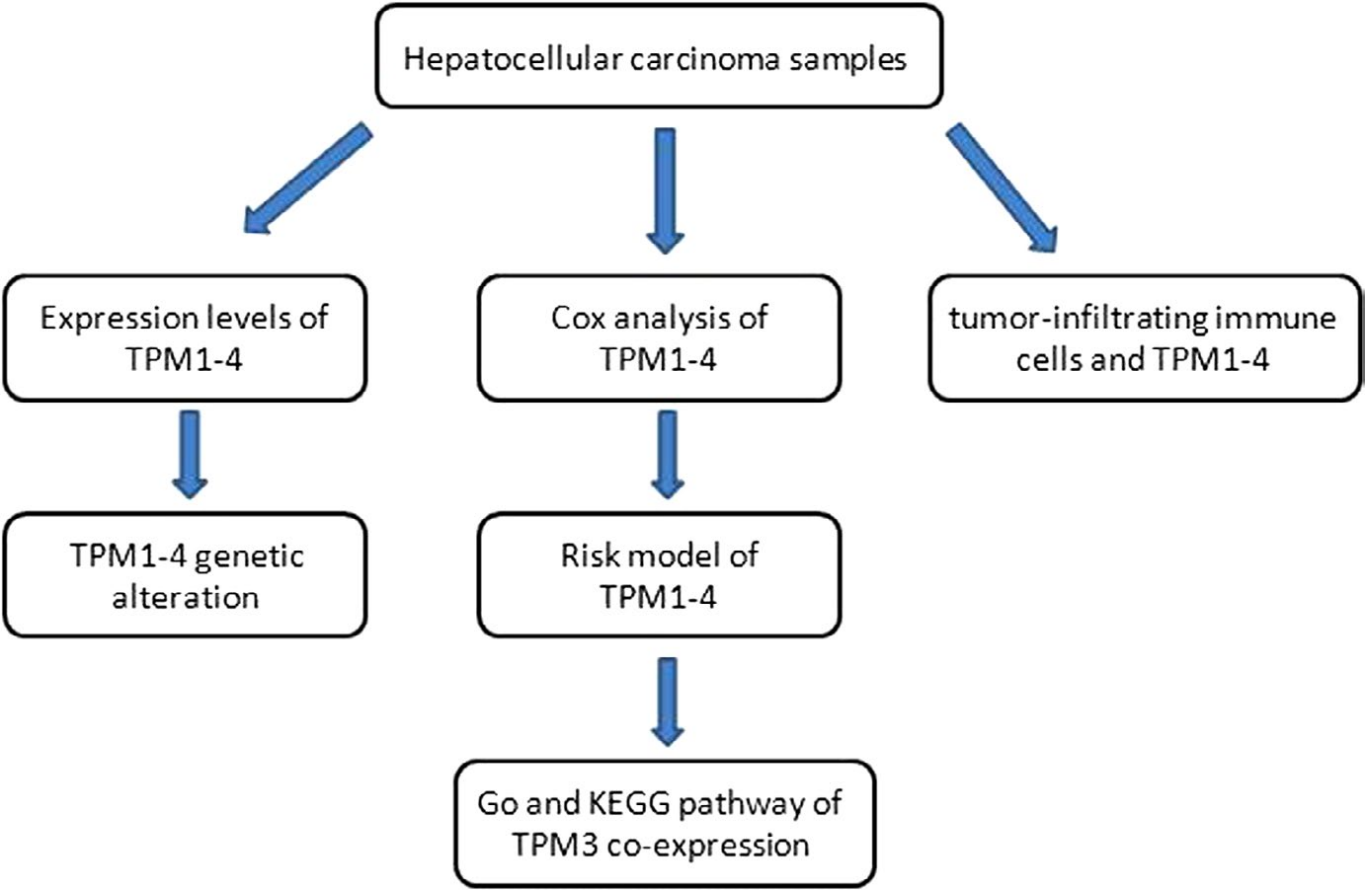
Flowchart of the study procedures

The procedures used in our study are illustrated in the flow chart as shown in Figure 1. Original TPM1–4 mRNA expression of TCGA HCC data and GTEx was downloaded from UCSC XENA (https://xenabrowser.net/datapages/). The human tumor samples were acquired from the TCGA database, and human normal samples were obtained from TCGA and GTEx database. Furthermore, the public database Oncomine (https://www.oncomine.org/) and GSE46408 were downloaded from Gene Expression Omnibus (GEO: https://www.ncbi.nlm.nih.gov/geo/) were used for verification.

### 2.2 GEPIA2

GEPIA2 (http://gepia2.cancer‐pku.cn/#index) is used to integrate the clinical data acquired from 9736 tumors and 8587 normal samples from TCGA and GTEx projects.^23^ Clinical stages and survival analysis were conducted through the “Expression Analysis” module. The survival results were shown by Kaplan–Meier (KM) curves with hazard ratios and *p* values from a log‐rank test. The cutoff *p* value was set as 0.05 in Student's *t* test.

### 2.3 cBioportal

The cBioPortal (http://cbioportal.org) is an open‐access comprehensive web resource that can be used to interactively explore and visualize multidimensional cancer genomics data sets.^24^ We utilized cBioPortal to assess genomic profiles of TPM1–4 in HCC including genes correlations, gene mutations, putative copy number alterations from GISTIC, and mRNA Expression *z*‐ scores (RNASeq V2 RSEM) with a *z* score threshold of ±1.8, Pearson's correction was included. In addition, the association of genetic alterations in TPM1–4 with disease‐free survival (DFS), progression‐free survival (PFS), and overall survival (OS) of HCC patients was investigated using the cBioPortal tool. The *p* value of <0.05 was considered statistically significant.

### 2.4 Construction of TPM prognostic model

Univariate and multivariate Cox regression analyses were performed to identify the prognostic value of TPM1–4 in HCC. The prognostic feature of TPM1–4 was further assessed by LASSO Cox regression, and the most useful prognostic biomarkers were selected using the “glment” package in R software with 10‐fold cross‐validation in order to generate the minimum cross‐validated error.^25^, ^26^ Thereafter, the risk score model was conducted by integrating candidate genes regression coefficients. The risk score was calculated by the following formula: $\sum_{i}\text{Coefficient}\;\left(\text{mRNAi}\right)\times \text{expression}\;\left(\text{mRNAi}\right)$. Akaike information criterion (AIC) was used to further verify the role of TPM1–4 in HCC. The KM curves were employed to evaluate the association of different risk score groups with the OS of HCC. The *p* value of KM survival curves was calculated by log‐rank tests. The area under the receiver operating characteristic curve (AUC) was used to estimate the predictive efficiency of the risk score model in HCC. Statistical significance was considered at the level of *p* < 0.05.

### 2.5 LinkedOmics

LinkedOmics database (http://www.linkedomics.org) is a publicly available website for the comprehensive analysis of multi‐omics data from TCGA.^27^ The correlation of TPM3 with differentially expressed genes in HCC was analyzed statistically using Pearson's correlation coefficient through “Linked Finder” module of LinkedOmics. In addition, Gene Ontology (GO, Biological Process) and Kyoto Encyclopedia of Genes and Genomes (KEGG) pathways of TPM3 co‐expression genes in HCC were conducted by Gene Set Enrichment Analysis (GSEA) through “LinkInterpreter” module of LinkedOmics.^28^

### 2.6 Tumor‐infiltrating immune cells in HCC

We utilize the CIBERSORT algorithm to make reliable immune infiltration estimations.^29^ SIGLEC15, TIGIT, CD274, HAVCR2, PDCD1, CTLA4, LAG3, and PDCD1LG2 were selected to be immune checkpoint–relevant transcripts, and the expression values of these eight genes were extracted. The analysis method and R package were implemented by the R foundation for statistical computing (2020), version 4.0.3, and software packages ggplot2 and pheatmap. Besides, we used Spearman's correlation analysis to describe the correlation between TPM1–4 and tumor mutational burden (TMB) or microsatellite instability (MSI). A *p* value of <0.05 was considered statistically significant. As an updated version of TIMER, TIMER2.0 (http://timer.cistrome.org/) is a freely available web server that provides a comprehensive analysis of tumor‐infiltrating immune cells in more than 30 cancer types.^30^ Here, we assessed the relevance of TPM1–4 to immune cells including B cell, CD4+ T cell, CD8+ T cell, macrophage, neutrophil, and dendritic cell in HCC.

## 3 RESULTS

### 3.1 TPM1–4 mRNA expression levels in HCC

**FIGURE 2:**
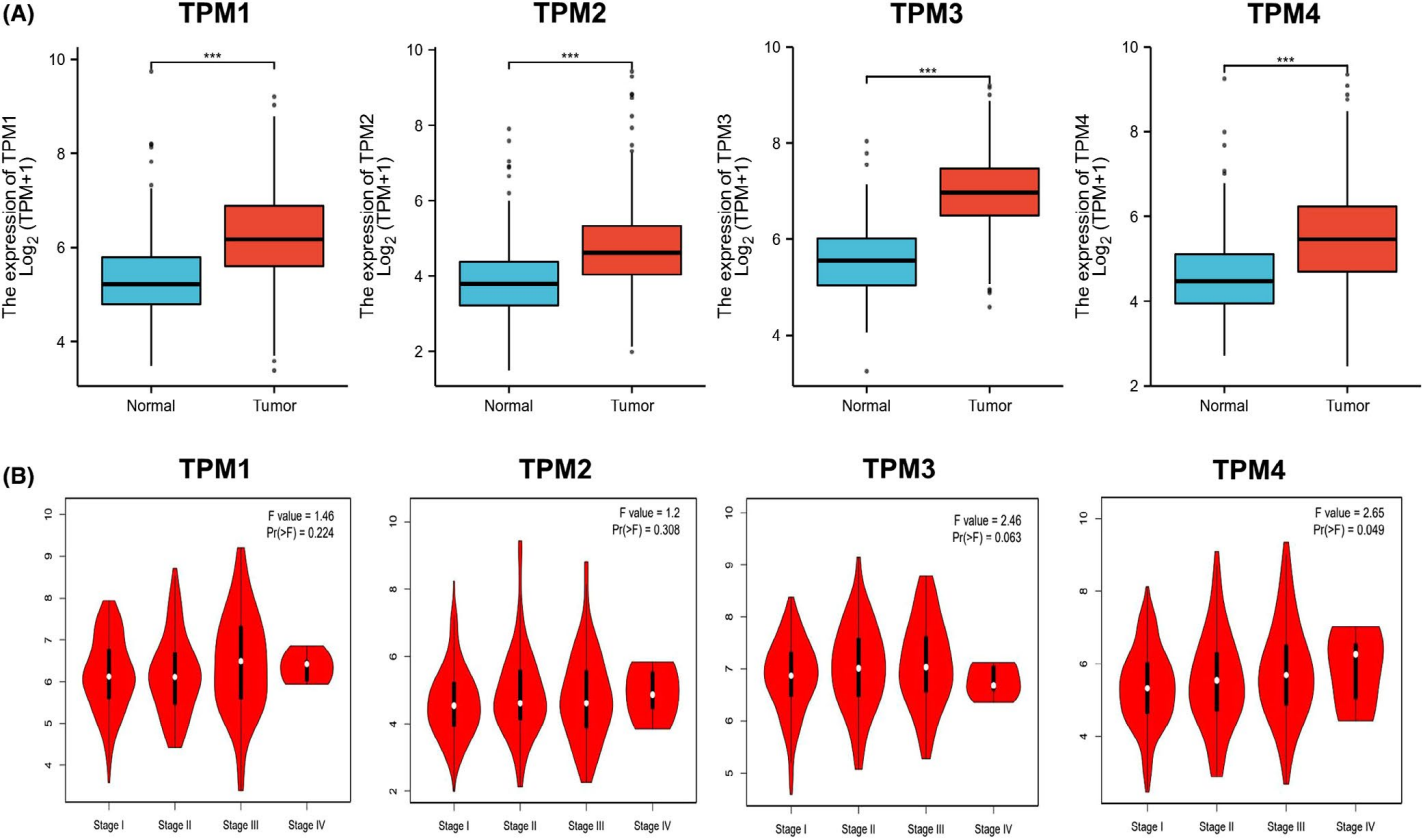
TPM1‐4 expression in HCC. (A) TPM1–4 expression in HCC tissues and normal tissues from TCGA and GTEx data (***p < 0.001). (B) The correlation between TPM1–4 and clinical stage in HCC patients (GEPIA2). HCC, hepatocellular carcinoma; TPM, tropomyosin

**FIGURE 3:**
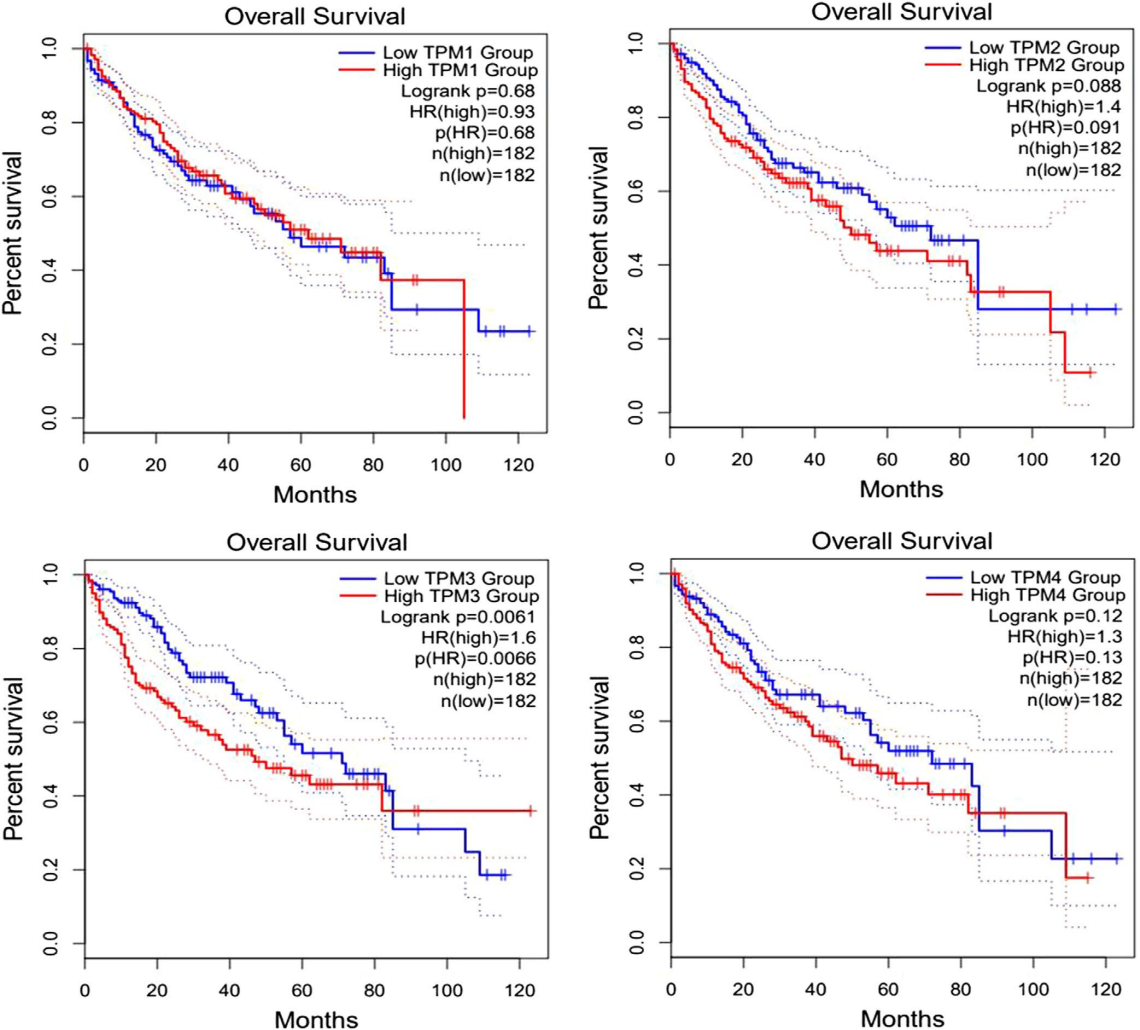
The correlation between TPM1–4 and OS in HCC (GEPIA2). HCC, hepatocellular carcinoma; OS, overall survival; TPM, tropomyosin

First, we assessed the expression of TPM1–4 in HCC from the TCGA and GTEx databases. Remarkably, TPM1–4 expression was all higher in HCC tissues in comparison with that in normal tissues (Figure 2A). Meanwhile, we investigated the correlation between TPM1–4 and the clinical stage in HCC patients (Table S1). GEPIA2 analytical results indicated that TPM4 groups significantly varied, whereas TPM1, TPM2, and TPM3 groups did not markedly differ (Figure 2B). Furthermore, we validated the mRNA expression of TPM1–4 in HCC from the Oncomine database and GSE46408. The results confirmed that TPM1–4 expression was all elevated in HCC tissues (Figure S1). KM survival analysis displayed high mRNA expression of TPM3 was notably related to worse OS of HCC patients. However, there were no associations between the mRNA expression of TPM1, TPM2, TPM4, and OS (Figure 3).

### 3.2 Genetic alteration of TPM1–4 in HCC

**FIGURE 4:**
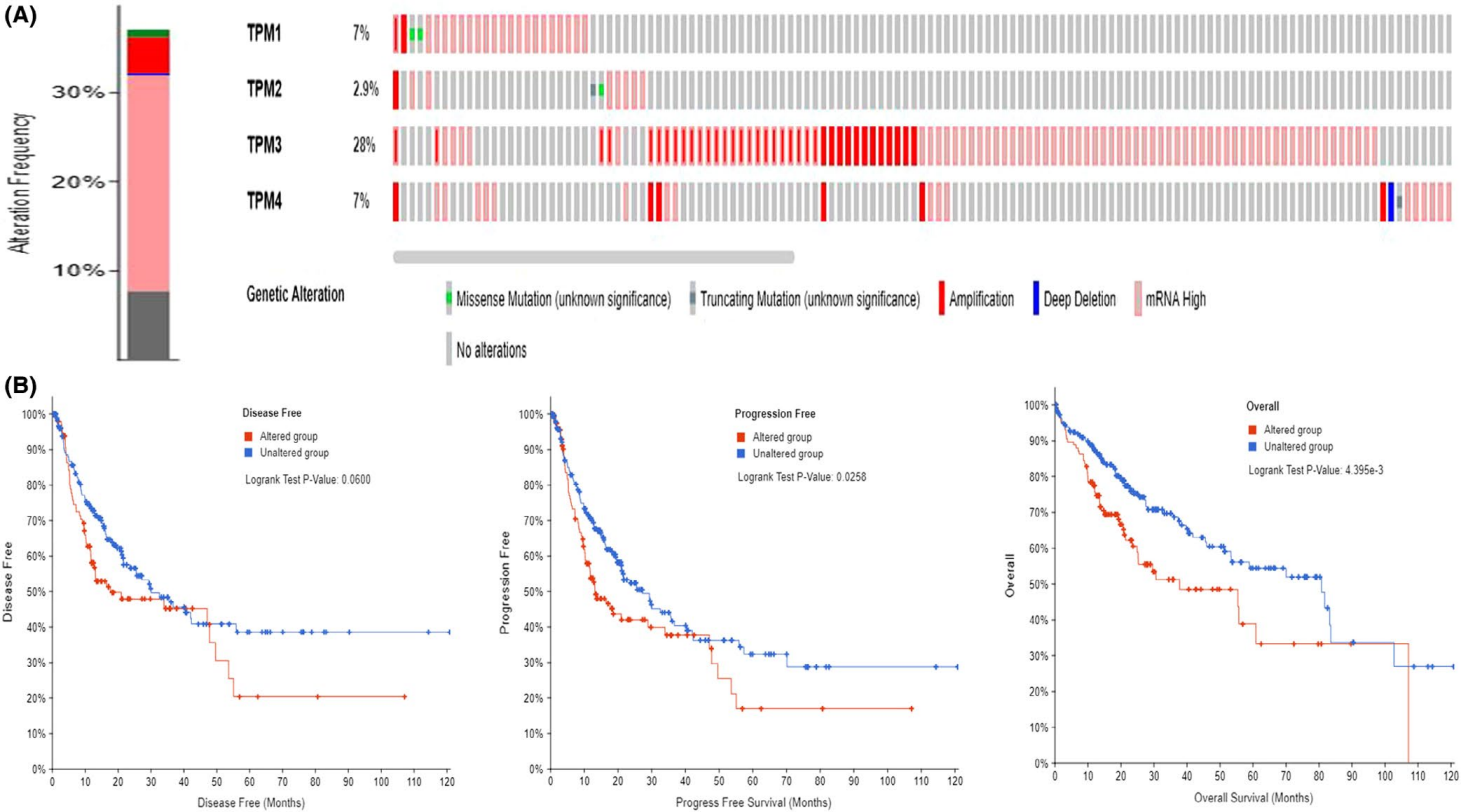
Genetic alteration of TPM1–4 in HCC. (A) Frequency of gene alterations in TPM1–4 in HCC. (B) Genetic alterations in TPM1–4 were related to shorter PFS (*p* < 0.05) and OS (*p* < 0.01) of HCC patients, no significant differences in DFS. DFS, disease‐free survival; HCC, hepatocellular carcinoma; OS, overall survival; PFS, progression‐free survival; TPM, tropomyosin

Next, we explored genetic variations of TPM1–4 in 348 HCC patients using the cBioportal database (TCGA, PanCancer Atlas). The results demonstrated genetic variations of TPM1–4 occurred in 129 (37%) of queried HCC patients, mRNA high and amplification were the most common altered types. Besides, the percentages of genetic variations ranged from 2.9% to 28% for individual genes (TPM1, 2.6%; TPM2, 6%; TPM3, 26%; TPM4, 6%) (Figure 4A). Genetic alteration of TPM1–4 was shown to be correlated with worse PFS (*p* = 0.0258) and OS (*p* = 0.004) of patients with HCC. However, there was no significant difference in DFS (*p* = 0.06) (Figure 4B).

### 3.3 Prognostic values of TPM1–4 in HCC

**FIGURE 5:**
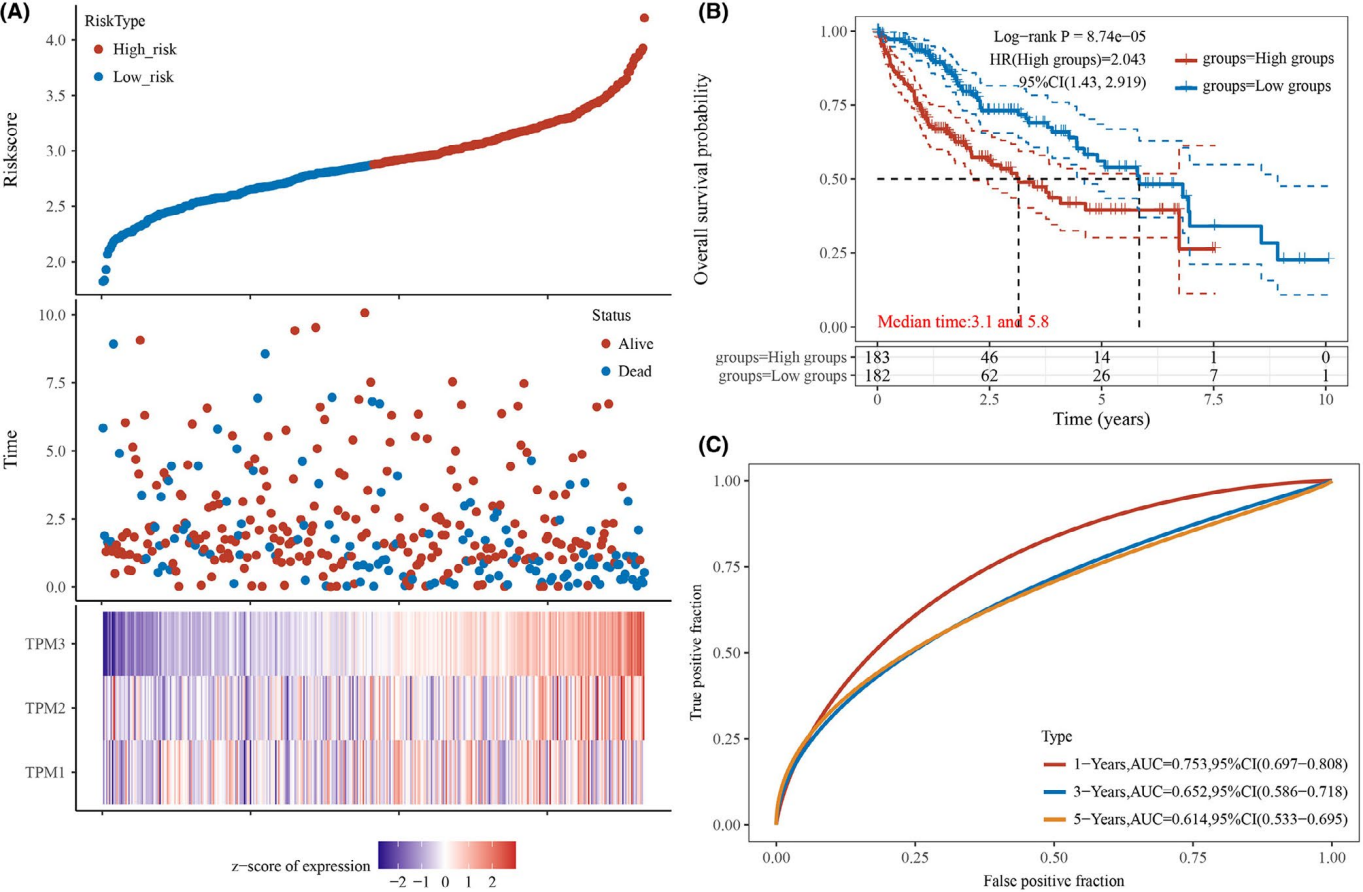
The prognostic values of the risk model in HCC. (A) The risk score of HCC patients in high‐ and low‐risk groups, the related genes expression was shown in heat maps. (B) Kaplan–Meier survival curve demonstrated that patients with high‐risk scores were correlated with worse overall survival. (C) The ROC curve of related genes with the AUC in HCC (0.753, 1‐year OS; 0.652, 3‐year OS; and 0.614, 5‐year OS). AUC, area under the curve; HCC, hepatocellular carcinoma; OS, overall survival; ROC, receiver operating characteristic

**FIGURE 6:**
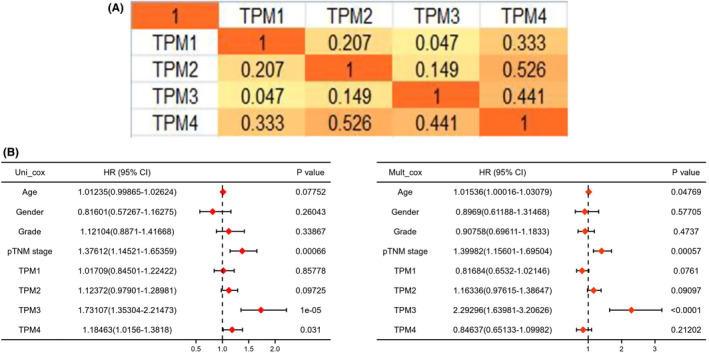
Univariate and multivariate Cox regression analyses of TPM1–4 and other clinicopathologic factors in HCC. (A) Correlation of TPM1–4 with each other in HCC (Spearman's correlation values, cBioPortal). (B) The forest plot for TPM1–4 and other clinicopathologic factors in HCC. HCC, hepatocellular carcinoma; TPM, tropomyosin

Subsequently, we evaluated the potential prognostic role of TPM1–4 in HCC by constructing a risk score model. And the clinical information of each patient was collected in Table S2, which contains age, sex, TNM stage, therapeutic regimen, drug use, OS, and so on. The risk score for each patient was calculated based on the following equation: Risk score=(‐0.1403)×TPM1+(0.0605)×TPM2+(0.5424)×TPM3. The analysis results exhibited the survival time of patients gradually decreased with the gradual increase of risk score. And the expression of related genes was shown in heat maps (Figure 5A). KM survival curve demonstrated patients with high‐risk scores were correlated with worse OS (Figure 5B). The areas under the ROC curve were 0.753 (1‐year OS), 0.652 (3‐year OS), and 0.614 (5‐year OS), demonstrating the prognosis prediction effectiveness of this risk model (Figure 5C). Additionally, we evaluated the performance of tumor stage and TNM at these three time points, respectively. The AUC of tumor grade corresponding to 1, 3, and 5 years was 0.487, 0.537, and 0.59 (Figure S2A). The AUC of the TNM stage corresponding to 1, 3, and 5 years was 0.671, 0.676, and 0.641 (Figure S2B). Moreover, we further explored the role of TPM1–4 in HCC according to the AIC. The results showed that the combination of TPM1 and TPM3 has relatively lower AIC values compared with other combinations (Table S3). Subsequently, we calculated the AUC of the top two combinations. The areas under the ROC curve of TPM1 and TPM3 were 0.744 (1‐year OS), 0.662 (3‐year OS), and 0.611 (5‐year OS) (Figure S3A). The areas under the ROC curve of TPM1, TPM3, and TPM4 were 0.746 (1‐year OS), 0.66 (3‐year OS), and 0.612 (5‐year OS) (Figure S3B). The above data demonstrated the prognosis prediction effectiveness of this risk model (TPM1, TPM2, and TPM3). The correlation analysis displayed significant and positive correlations between TPM1 and TPM2, TPM1 and TPM4, TPM2 and TPM3, TPM2 and TPM4, and TPM3 and TPM4 (Figure 6A). Besides, we observed that clinical stage (pTNM) and TPM3 are significantly correlated with the OS of HCC, which implied lower clinical stage and decreased TPM3 expression may be independent prognostic factors of a favorable prognosis (Figure 6B).

### 3.4 TPM3 co‐expression networks in HCC

**FIGURE 7:**
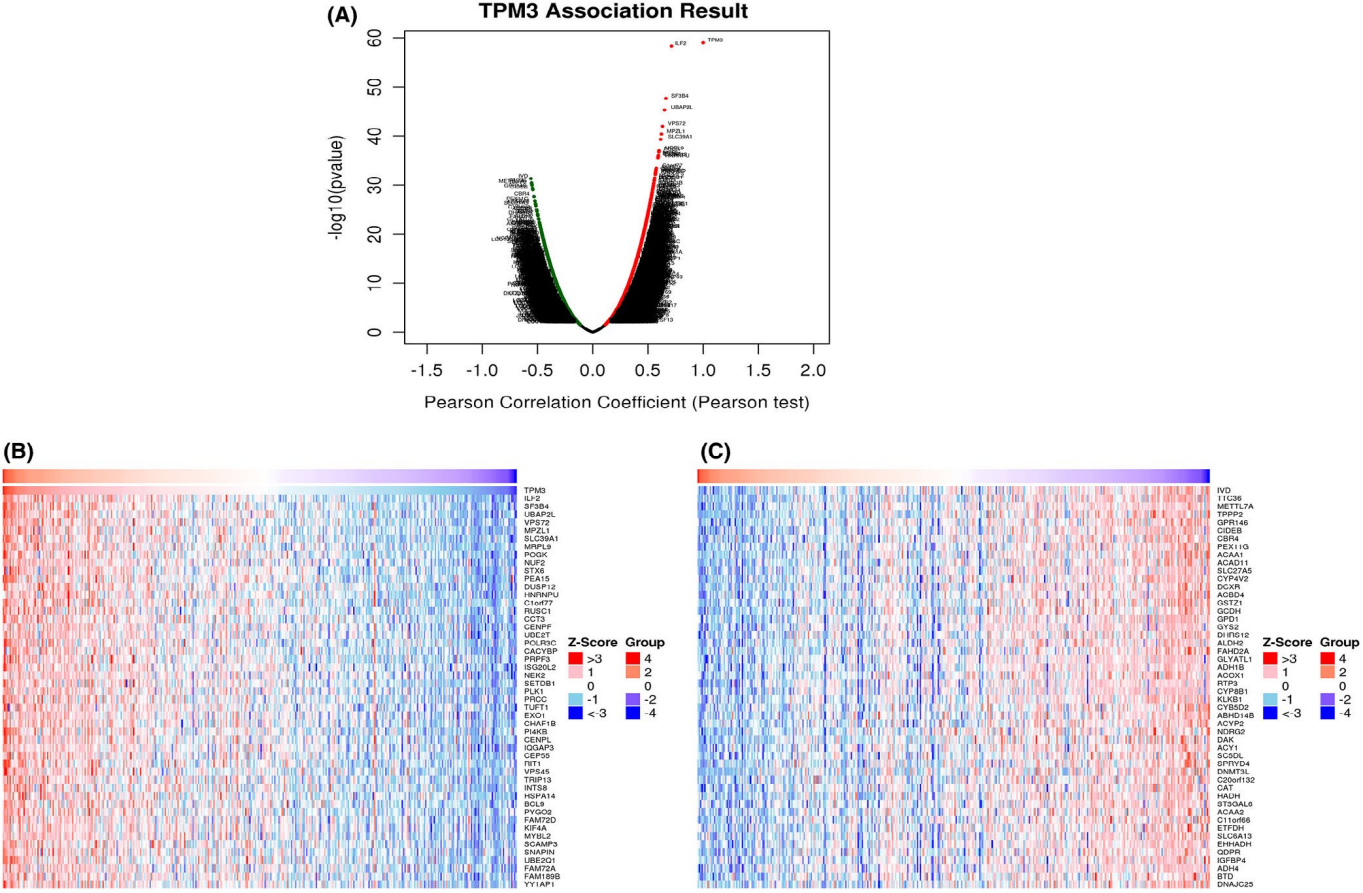
TPM3 co‐expression genes in HCC (LinkedOmics). (A) The Pearson test was used to identify genes differentially expressed in correlation with TPM3 in HCC. (B, C) Heat map demonstrated 50 significant genes positively and negatively correlated with TPM3 in HCC. HCC, hepatocellular carcinoma; TPM, tropomyosin

**FIGURE 8:**
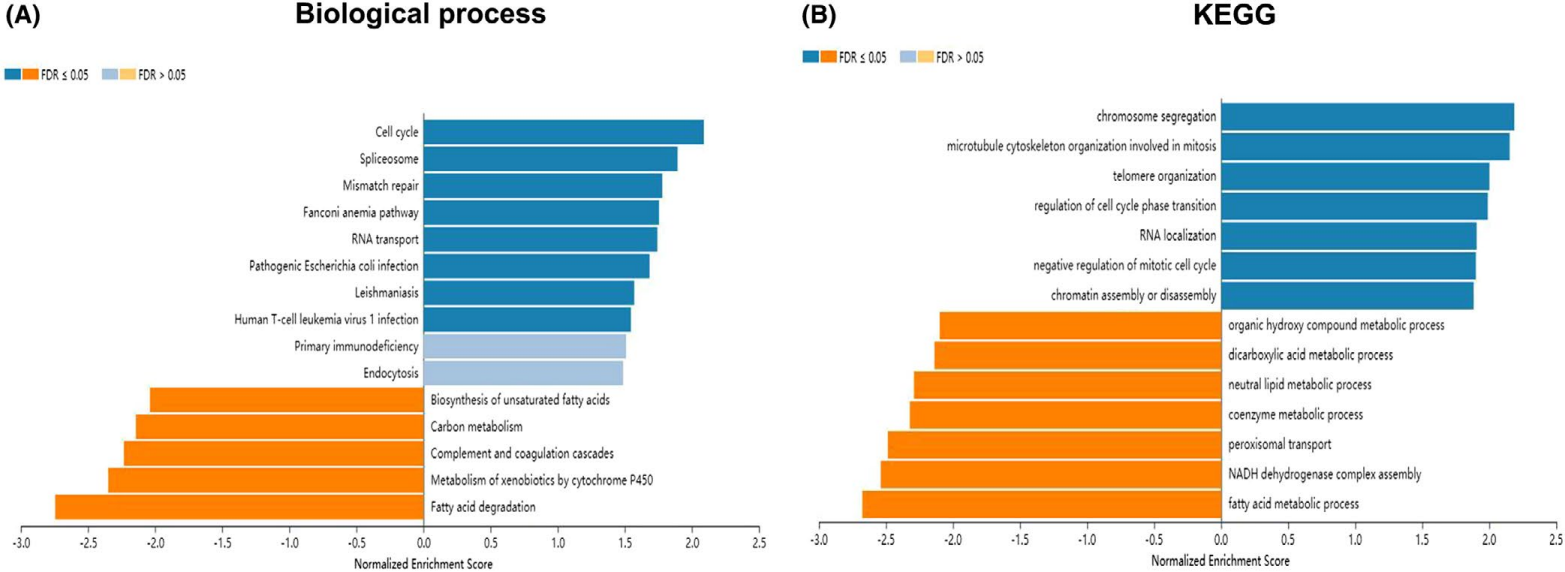
GO (biologic process) terms and KEGG pathway related to TPM3 in HCC (LinkedOmics). (A) The significantly enriched GO annotations (biologic process) of TPM3 in HCC. (B) The KEGG pathways of TPM3 in HCC. GO, Gene Ontology; HCC, hepatocellular carcinoma; KEGG, Kyoto Encyclopedia of Genes and Genomes; TPM, tropomyosin

The above data suggested TPM3 may play a key role in HCC development, thus we utilized LinkedOmics to analyze the co‐expressed genes of TPM3 in 371 HCC patients. There were 13,260 genes (red dots) positively correlated with TPM3, and 6661 genes (green dots) negatively correlated with TPM3 (Figure 7A). The heat map demonstrated 50 significant genes positively and negatively correlated with TPM3 (Figure 7B,C). Significant GO term annotation was carried out by GSEA. And TPM3 co‐expression genes mainly participated in chromosome segregation, microtubule cytoskeleton organization involved in mitosis, spindle organization, and cell cycle checkpoint, while fatty acid metabolic process, small molecular catabolic process, peroxisomal transport, mitochondrial respiratory chain complex assembly, and peroxisome organization were inhibited (Figure 8A). KEGG pathway analysis manifested these genes were primarily involved with cell cycle, DNA replication, spliceosome, mismatch repair, and RNA transport (Figure 8B).

### 3.5 Association between TPM1–4 and tumor‐infiltrating immune cells in HCC

**FIGURE 9:**
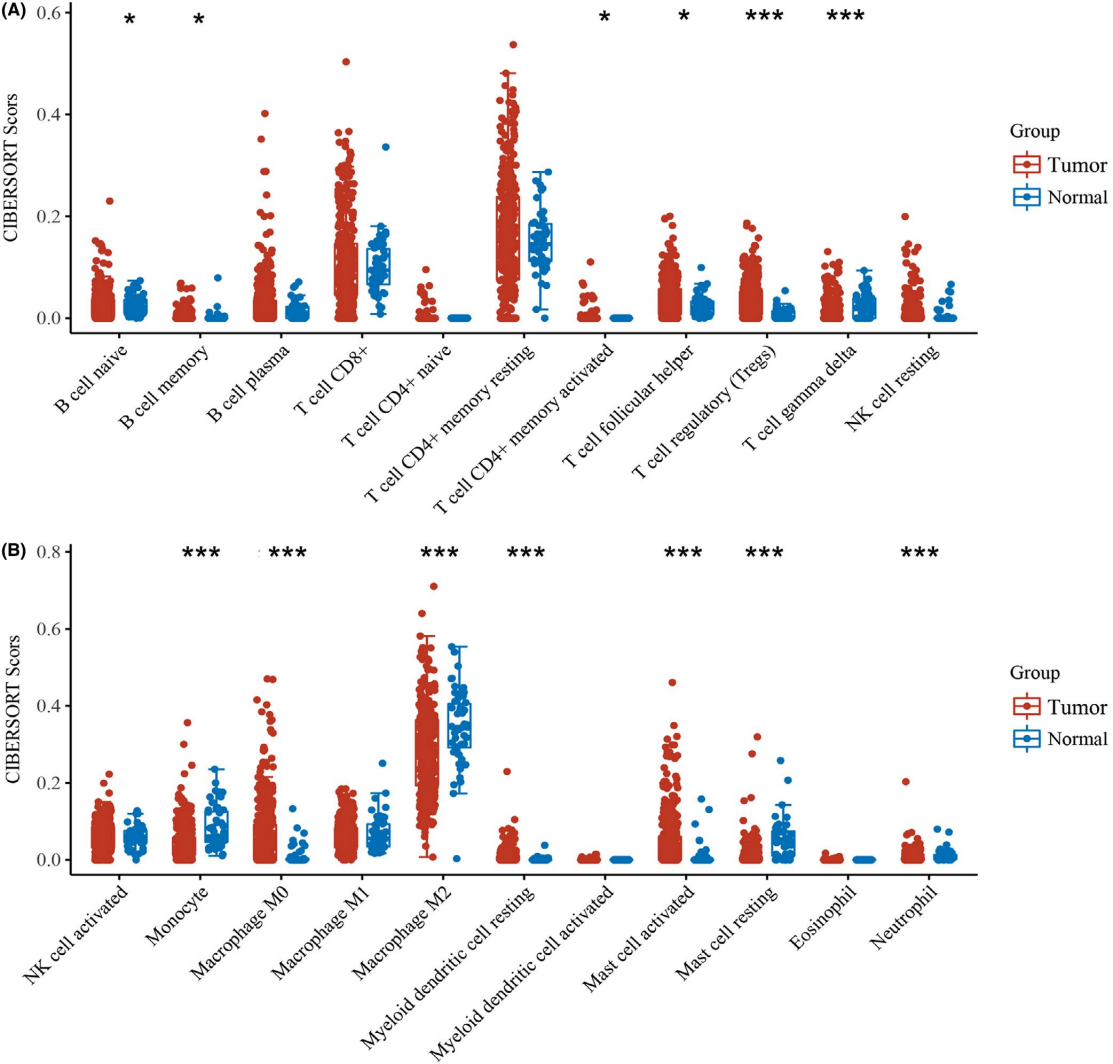
The score distribution of tumor‐infiltrating immune cells in HCC. Twenty‐two kinds of tumor‐infiltrating immune cells CIBERSORT score distribution in HCC tissues and normal tissues (*p < 0.05, ***p < 0.001)

**FIGURE 10:**
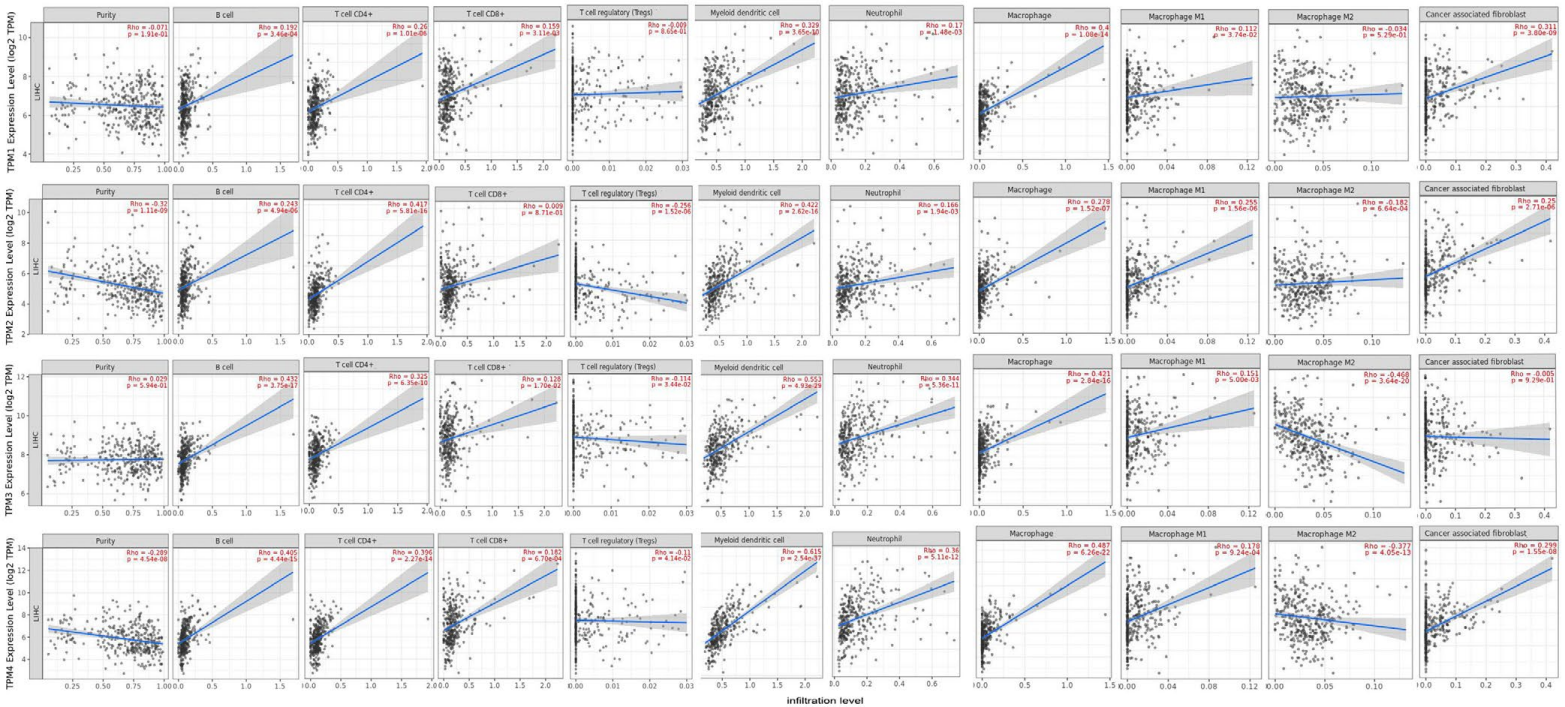
Association between TPM1–4 and tumor‐infiltrating immune cells in HCC (TIMER2.0). Tumor purity was shown on the left panel. The correlation between TPM1–4 and tumor‐infiltrating immune cells (B cell, CD4+ T cell, CD8+ T cell, Tregs, macrophage, neutrophil, dendritic cell, and cancer‐associated fibroblast) were shown in HCC. HCC, hepatocellular carcinoma; TPM, tropomyosin

In the last decade, an increasing amount of data has pointed to a key role for tumor‐infiltrating lymphocytes in human cancers.^31^ Thus we estimated the score distribution of tumor‐infiltrating immune cells in HCC using CIBERSORT algorithm and the results were shown in Figure 9. Besides, most immune checkpoints‐related genes expression were significantly upregulated in HCC tissues compared with that in normal tissues except for LAG3 and PDCD1LG2 (Figure S4). Spearman's correlation analysis results showed that the association between TPM4 and TMB (partial‐cor = −0.27, *p* < 0.001) were comparably low (Figure S5), and there is no significant association between TPM1–4 and MSI (Figure S6). Afterward, we studied the relationship between TPM1–4 and tumor‐infiltrating immune cells in HCC using the TIMER2 database. Our results demonstrated that the expression of TPM2 and TPM4 were negatively correlated with tumor purity (*p* < 0.05) indicating highly expression in HCC microenvironment. Moreover, the association between B cell and TPM3 (partial‐cor =0.432, *p* < 0.001), CD4+ T cell and TPM2 (partial‐cor =0.417, *p* < 0.001), CD8+ T cell and TPM4 (partial‐cor =0.182, *p* < 0.001), dendritic cell and TPM4 (partial‐cor =0.615, *p* < 0.001), neutrophil and TPM4 (partial‐cor =0.36, *p* < 0.001), macrophage and TPM4 (partial‐cor =0.487, *p* < 0.001), macrophage M1 and TPM2 (partial‐cor =0.255, *p* < 0.001), macrophage M2 and TPM3 (partial‐cor = −0.468, *p* < 0.001), and cancer‐associated fibroblast and TPM2 (partial‐cor =0.311, *p* < 0.001) were comparably high (Figure 10).

## 4 DISCUSSION

Over the past decade, there have been great advancements in clinical diagnosis and management for HCC. Although imaging examinations such as ultrasound, computed tomography, magnetic resonance imaging, and serum alpha‐fetoprotein have been widely applied to surveillance of HCC, the early diagnosis rate of HCC is still not high.^32^ Currently, surgical resection is the primary treatment modality for HCC, combined with chemotherapy, radiotherapy, molecule targeted therapy, interventional therapy, and Chinese medicine treatment.^33^ Despite the progress in comprehensive treatment techniques, the prognosis of HCC remains poor with a 5‐year survival rate of <20% which is mainly attributed to a high recurrence rate.^3^, ^34^ Therefore, it is necessary to search for potential biomarkers to improve the prognosis of patients with HCC.

Physiologically, the actin cytoskeleton plays a crucial role in the cellular process involving cell proliferation, migration, apoptosis, and differentiation.^35^, ^36^, ^37^ Altered expression of actin‐binding proteins can result in dysregulation of the actin cytoskeleton, which is associated with oncogenic transformation.^38^, ^39^ The cytoskeletal tropomyosins belong to actin‐binding proteins and can stabilize actin filaments during the process of actin assembly.^40^ Although dysregulation of TPM1–4 expression may be related to the pathogenesis of some cancers, a comprehensive analysis of TPM1–4 in HCC is yet to be characterized.

TPM1 (also known as α‐tropomyosin) is a widely expressed actin‐binding protein, which is involved in molecular communication on cellular surface and intercellular proliferation signaling among normal cells.^41^ Dysregulation of TPM1 expression causes stress fiber disruption, resulting in changes in cell morphology and motility, thereby leading to malignant transformation of normal cells.^42^ Bharadwaj and Prasad first reported the level of TPM1 expression declined in breast cancer.^14^ Subsequent studies have also shown that TPM1 may function as an anti‐oncogene to suppress many types of cancer development including gastric cancer, bladder carcinoma, lung cancer, and intrahepatic cholangiocarcinoma.^43^, ^44^, ^45^, ^46^ TPM2 (also called β‐tropomyosin) is expressed primarily in fibroblasts, smooth, and skeletal muscle cells. In the normal physiologic state, TPM2 mainly participated in the regulation of cell motility and muscle contraction.^47^ Aberrant TPM2 expression is known to contribute to a series of rare myopathies.^48^ Recent studies also revealed that TPM2 can exert pro‐ or antitumorigenic roles in cancers, which may be related to the functional specificity of the gene in different kinds of cancers. TPM3 (namely γ‐tropomyosin) mediates the response of myosin to calcium ions and maintains the stability of cytoskeletal microfilaments in skeletal muscle cells.^49^ Some evidence suggests that TPM3 in nonmuscle tissues may take part in tumors progression. Kim et al. found that overexpression of TPM3 could significantly increase the risk of HCC.^50^ Choi et al. confirmed that TPM3 amplification facilitated the epithelial–mesenchymal transition and downregulated the expression of epithelial cadherin, which finally induced HCC cells proliferation and invasion.^51^ These studies implied that high expression of TPM3 may promote HCC progression. TPM4 (also referred to as δ‐tropomyosin), first discovered in *Xenopus* embryos, regulated the contraction of skeletal and smooth muscle cells or maintained the stability of the cytoskeleton in nonmuscle cells.^52^, ^53^, ^54^ Previous research reported that TPM4 is related to the occurrence and metastasis of several cancers. Dube et al. first observed that high expression of TPM4 existed in ovarian cancer.^55^ Similarly, increased TPM4 expression promoted HCC cell proliferation and invasion which may be applied as a diagnostic and prognostic marker for HCC development.

In the present study, we found TPM1, TPM2, TPM3, and TPM4 were all upregulated in HCC tissues. Although high expression of TPM3 was correlated with poor survival outcomes in patients with HCC. Besides, we observed that TPM3 amplification was the most common altered type in TPM1–4 genetic alteration, which was associated with unfavorable PFS and OS of HCC. Additionally, the risk model revealed that TPM1, TPM2, and TPM3 were applied to risk assessment of HCC prognosis, among which TPM1 exert the negative coefficient. Upregulation of TPM1 may suppress the development of some cancers by inhibiting cellular morphologic transformation.^56^ That means the high expression of TPM1 may act as a good prognostic factor in HCC development which is consistent with previous studies. Moreover, TPM3 expression was significantly higher in the high‐risk group than that in the low‐risk group. Univariate and multivariate Cox regression analyses indicated that TPM3 may be an independent prognostic factor of HCC prognosis. These data implied that TPM3 may play more important roles in HCC development compared with the other three genes. Given that the critical role of TPM3 in HCC, we explored the potential molecular mechanism of TPM3 by building TPM3 co‐expression networks in HCC. Consistent with previous studies, TPM3 co‐expression genes mainly participated in the cell cycle by maintaining microtubule cytoskeleton in HCC progression.

The tumor microenvironment is increasingly recognized to play an integral role in HCC progression. In addition to tumor cells, the liver tumor microenvironment primarily comprises tumor‐infiltrating lymphocytes, tumor‐associated macrophages, tumor‐associated neutrophils, cancer‐associated fibroblasts, myeloid‐derived suppressor cells, dendritic cells, extracellular matrix, and other matrix‐associated molecules.^57^ The different score distribution of tumor‐infiltrating immune cells and immune checkpoints‐related genes expression in HCC further confirmed that tumor microenvironment affects HCC progression. CD8+ cytotoxic T lymphocytes can efficiently kill tumor cells by secreting cytokines which are associated with a good prognosis.^58^ Regulatory T cells, a suppressive subset of CD4+ T lymphocytes, can suppress immune reactions induced by CD8+ cytotoxic T lymphocytes to promote tumor escape.^59^ B cells can exert antitumor effects through antibody production and by serving as antigen‐presenting cells to induce T cell responses.^60^ TPM3 was positively correlated with B cells, TPM2 was positively associated with CD4+ T cells and negatively associated with Tregs, and TPM4 was positively associated with CD8+ T cells. Given that TPMs were involved in the almost whole cellular process, these data suggested that the upregulation of TPMs in HCC may recruit inactivated B and T cells to tumor sites. Once activated by drugs or other treatments, these lymphocytes could suppress HCC development. Tumor‐associated neutrophils are capable of recruiting macrophage and regulatory T cells into tumor sites by releasing cytokines to facilitate tumor progression and metastasis.^61^ Some molecular and cellular components in the tumor microenvironment enable to cause functional abnormalities of dendritic cells and thus allowing tumor cells to escape immune surveillance.^62^ In hepatocellular carcinoma, cancer‐associated fibroblasts are involved in crosstalk between tumor cells by activating various pathways (signaling pathways) and initiating the expression of various cytokines, thus providing a microenvironment suitable for the development of tumor cells.^63^ TPMs were positively associated with these cells implying TPMs were likely to promote HCC progression by stimulating the oncogenic ability of these cells. Tumor‐associated macrophages are divided into the tumor growth‐inhibiting M1 and the tumor‐promoting M2 phenotype.^64^ M1‐like macrophages may inhibit HCC development by changing tumor microenvironment, and M2‐like macrophages can promote HCC cell proliferation and invasion through activating TLR4/STAT3 signaling pathway.^65^, ^66^ TPM2 was positively correlated with macrophage M1 and TPM3 was negatively correlated with macrophage M2 which means that TPMs may promote macrophage M2 polarization to exert an antitumor effect on HCC progression. The above data demonstrated that TPM1–4 plays complex immunological roles in the tumor microenvironment and further research was needed to explore the possible mechanism. In short, we elucidated the role of TPM1–4 in HCC progression through bioinformatic comprehensive analysis. However, the theoretical analysis and limited sample size may bias the results. Experimental validation in vivo and in vitro needs to be further studied.

## 5 CONCLUSION

Our study detected that the expression of TPM1–4 was all significantly upregulated in HCC, suggesting TPM1–4 may serve as an important role in HCC development. High TPM3 expression was found to be associated with poor OS, and TPM3 may be an independent prognostic factor for HCC. The risk assessment model identified high‐risk groups of HCC patients. These analysis results might provide new therapeutic strategies for the diagnosis and treatment of HCC.

## CONFLICT OF INTEREST

All authors declare that they have no conflict of interest.

## AUTHOR CONTRIBUTIONS

Zhihui Tian and Yusheng Wang designed experiments; Yusheng Wang supervised experiments; Zhihui Tian and Jian Zhao drafted the manuscript; Zhihui Tian, Jian Zhao, and Yusheng Wang collected the data and performed data analysis. All authors read and approved the manuscript.

## Supporting information

Supplementary Material

## Data Availability

The data sets analyzed during the present study are available from online repositories: UCSC XENA, Oncomine database, GSE46408, GEPIA2, cBioportal, TIMER2.0 and LinkedOmics.
