# Supplementary material for: The prognostic value of TPM1–4 in hepatocellular carcinoma
Source: Cancer Med. 2021 Nov 30;11(2):433–46. doi: 10.1002/cam4.4453 (PMC8729055; doi:10.1002/cam4.4453)
Supplement: Supplementary file 1 — Supplementary Material [file CAM4-11-433-s001.zip › cam44453-sup-0010-Supinfo.docx]

**Supplement Figure legends:**

**Figure S1.** The mRNA expression of TPM1-4 in HCC from Oncomine database and GSE46408. A, The mRNA expression of TPM1-4 (cancer vs. normal tissue of liver) was assessed using the Oncomine database. Red represents significant overexpression and blue represents reduced expression. B: The mRNA expression of TPM1-4 from GSE46408 in volcano plot. Red dots represent upregulated genes and blue dots represent downregulated genes.

**Figure S2.** A, the AUC corresponding to 1, 3 and 5 years of tumor grade using K-M plot. B, the AUC corresponding to 1, 3 and 5 years of TNM stage using K-M plot.

**Figure S3.** A, the AUC corresponding to 1, 3 and 5 years of combination of TPM1 and TPM3 using K-M plot. B, the AUC corresponding to 1, 3 and 5 years of TPM1, TPM3 and TPM4 using K-M plot.

**Figure S4.** Immune checkpoints related genes expression in HCC. SIGLEC15, TIGIT, CD274, HAVCR2, PDCD1 and CTLA4 expression were significantly upregulated in HCC tissues compared to that in normal tissues (^*^*p*<0.05; ^**^*p*<0.01; ^***^*p*<0.001).

**Figure S5.** Association between TPM1-4 and TMB in HCC.

**Figure S6.** Association between TPM1-4 and MSI in HCC.
