# Supplementary figures and images for: The prognostic value of TPM1–4 in hepatocellular carcinoma
Source: Cancer Med. 2021 Nov 30;11(2):433–46. doi: 10.1002/cam4.4453 (PMC8729055; doi:10.1002/cam4.4453)

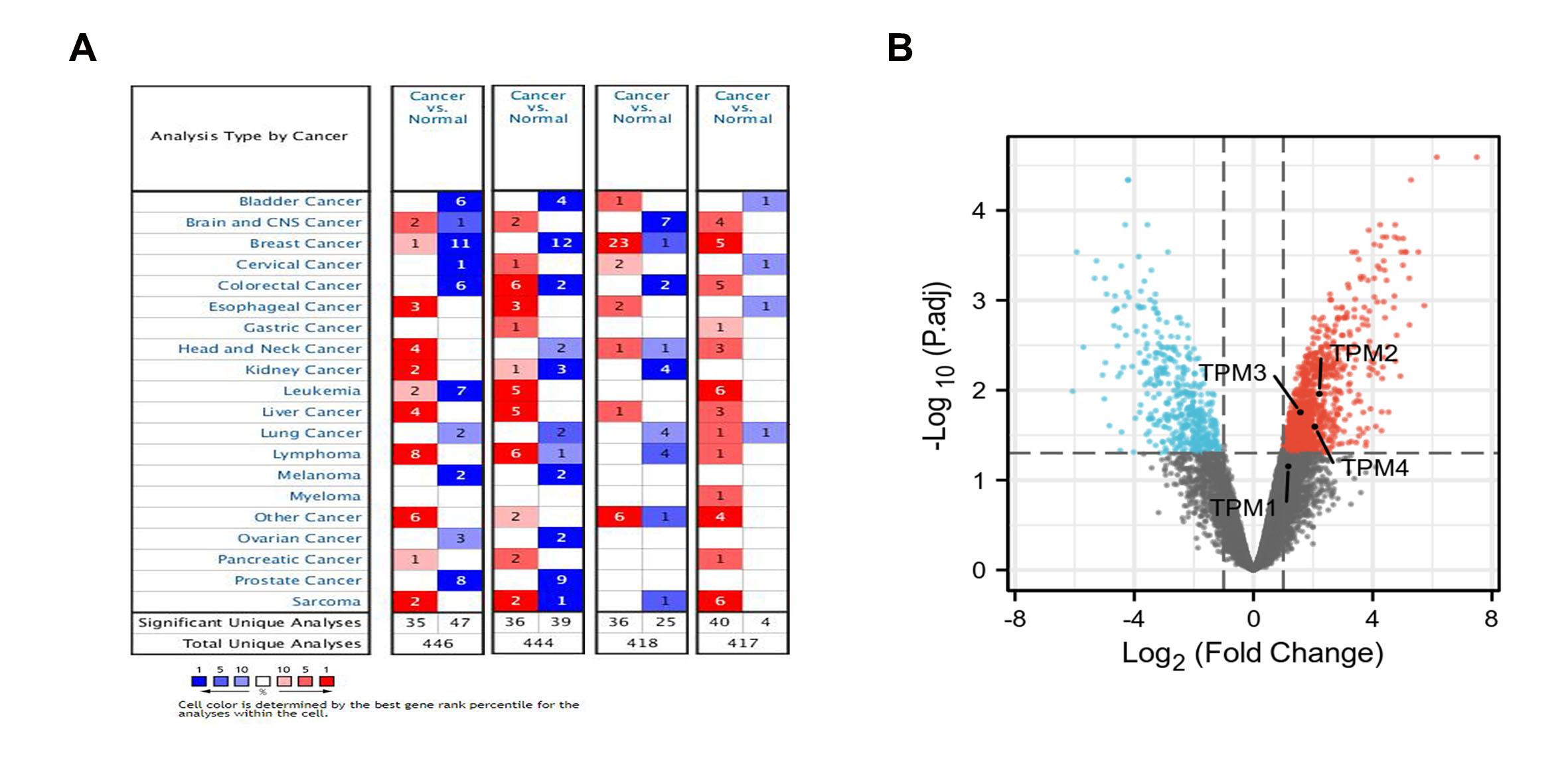

Supplement: Supplementary file 1 — Supplementary Material [file CAM4-11-433-s001.zip › cam44453-sup-0001-FigS1.jpg]

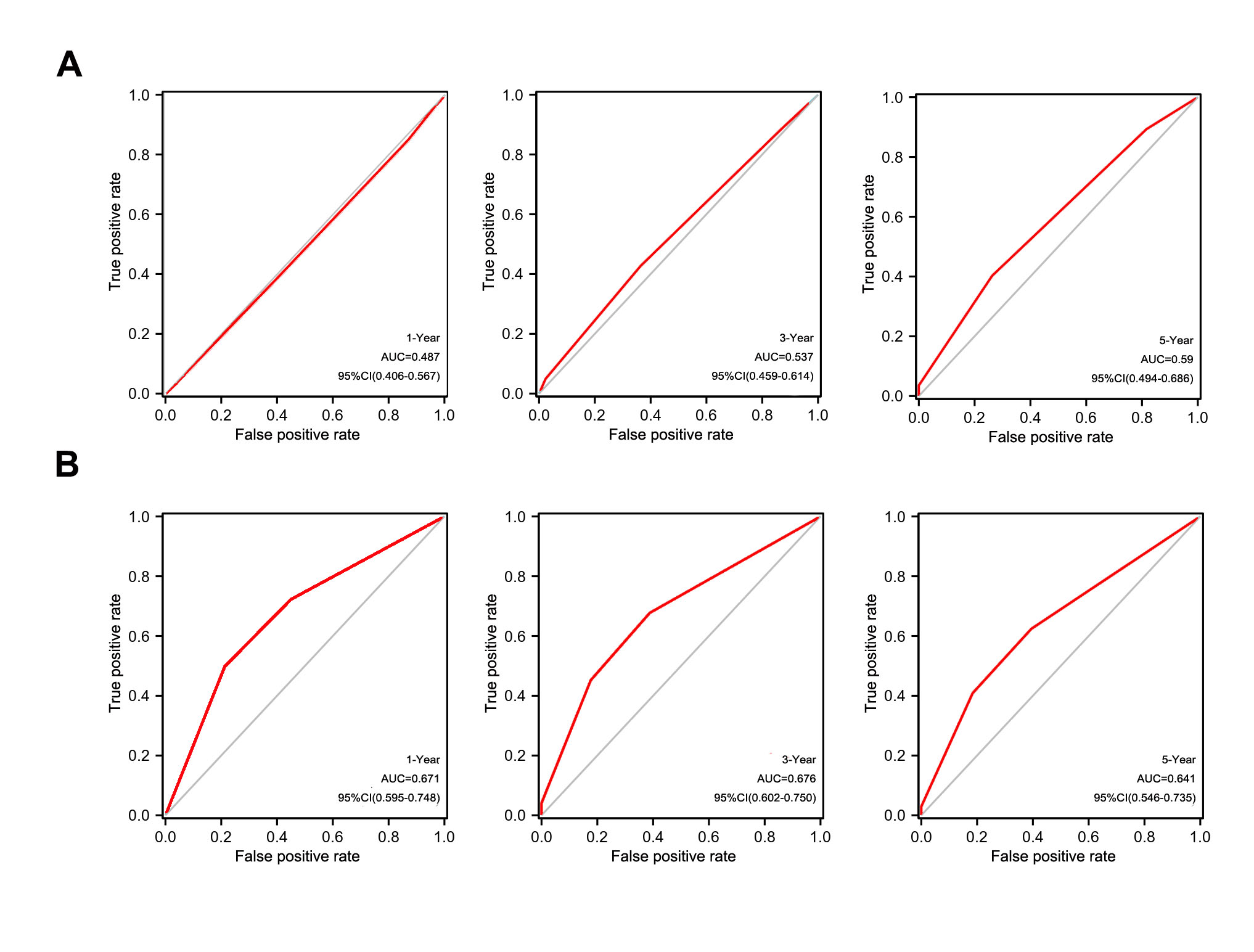

Supplement: Supplementary file 1 — Supplementary Material [file CAM4-11-433-s001.zip › cam44453-sup-0002-FigS2.jpg]

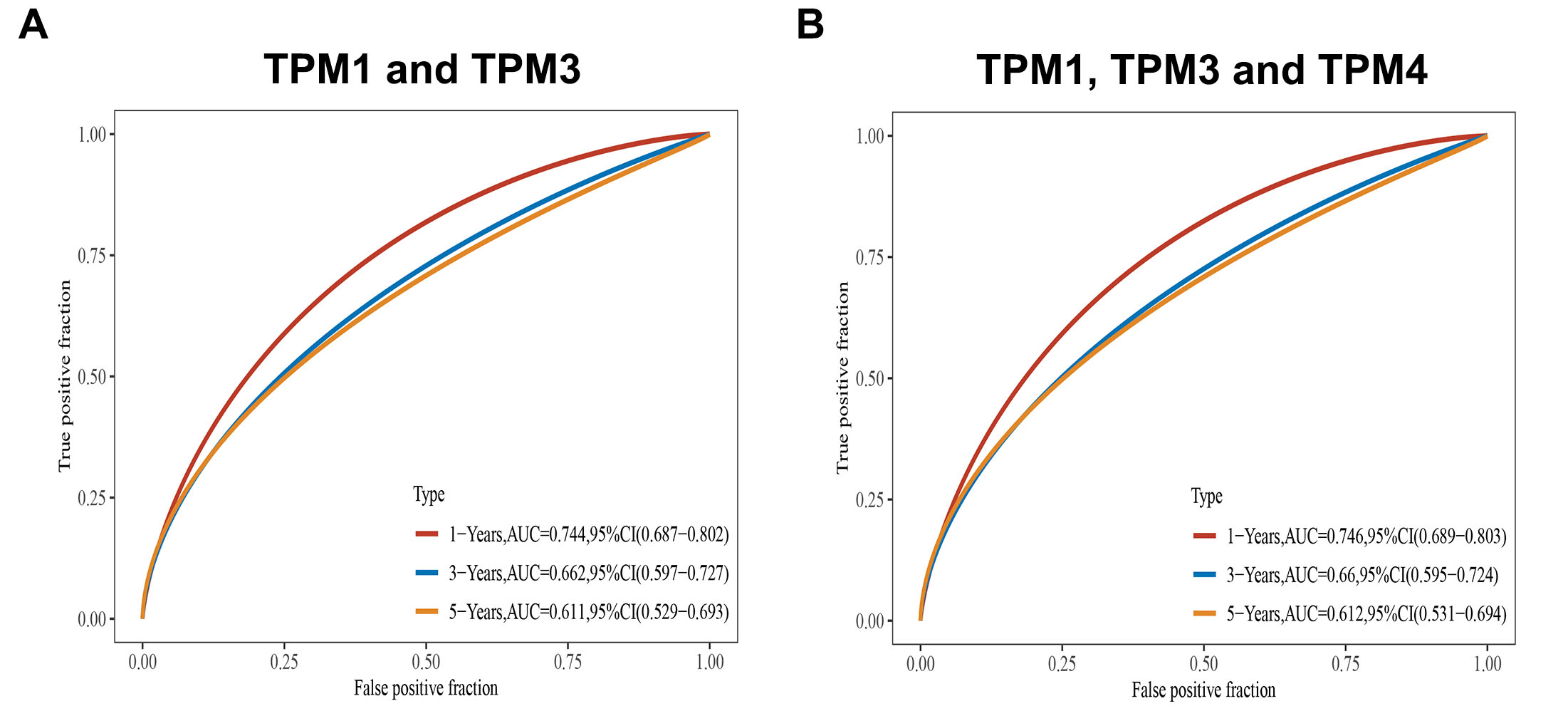

Supplement: Supplementary file 1 — Supplementary Material [file CAM4-11-433-s001.zip › cam44453-sup-0003-FigS3.jpg]

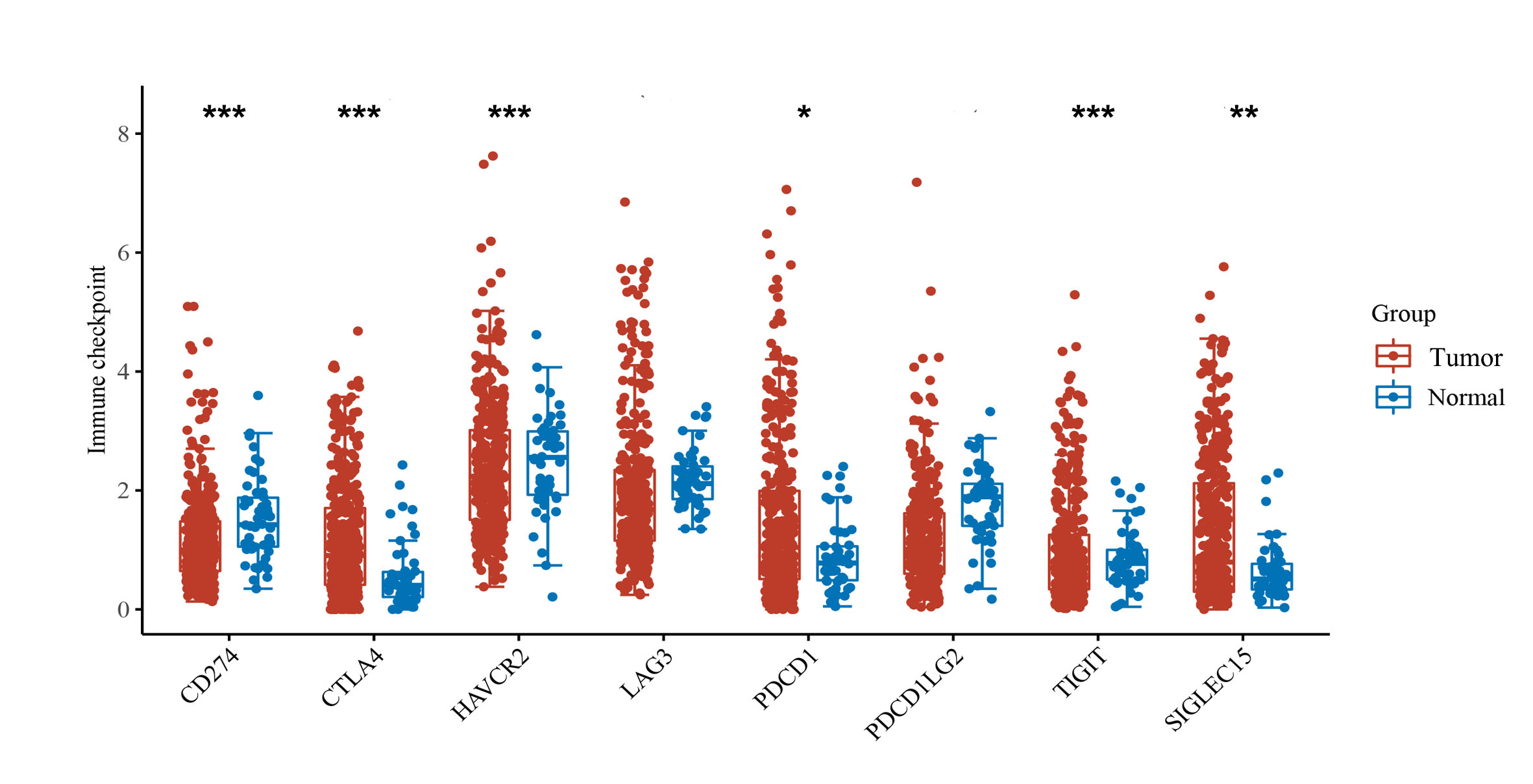

Supplement: Supplementary file 1 — Supplementary Material [file CAM4-11-433-s001.zip › cam44453-sup-0004-FigS4.jpg]

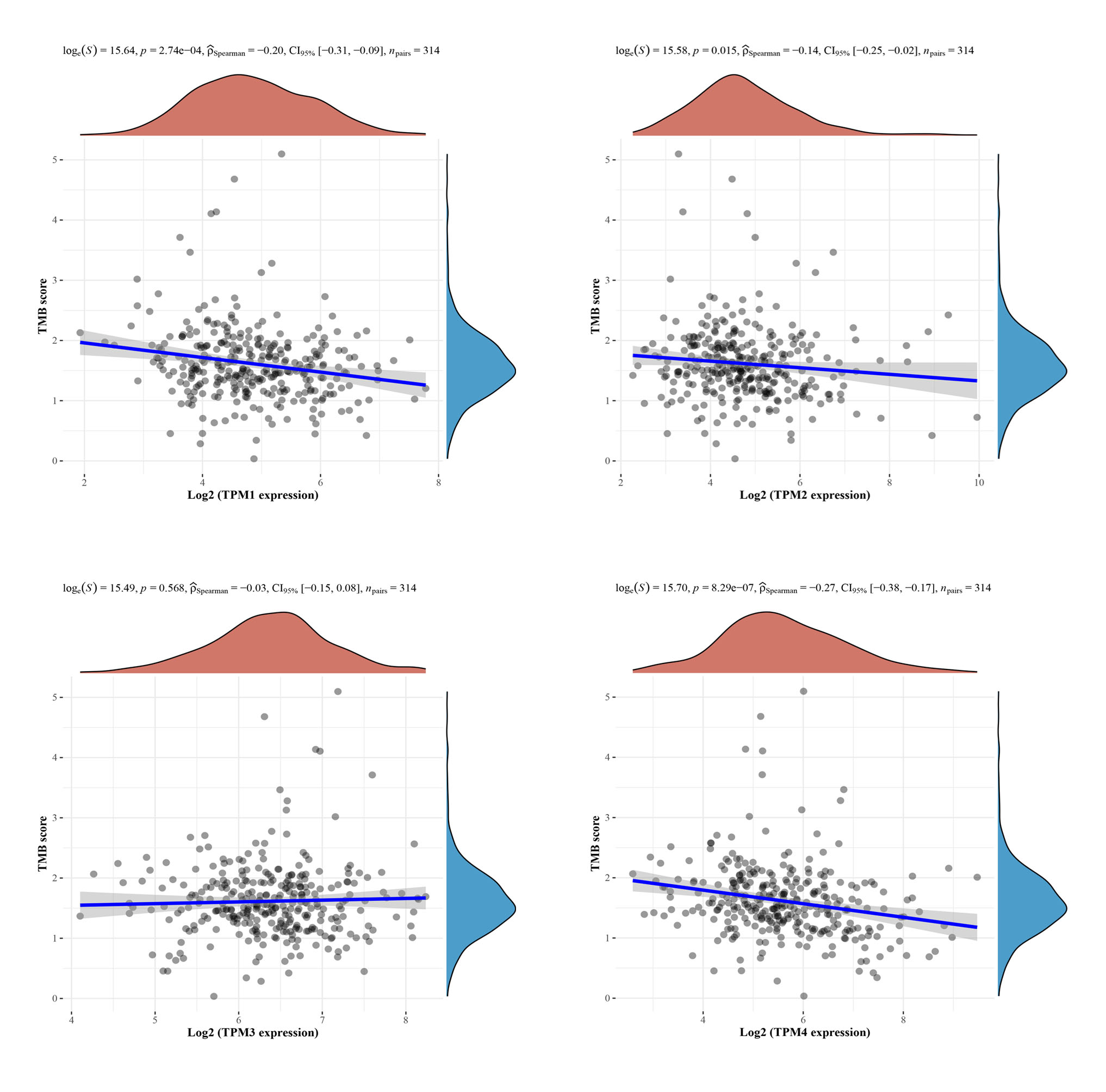

Supplement: Supplementary file 1 — Supplementary Material [file CAM4-11-433-s001.zip › cam44453-sup-0005-FigS5.jpg]

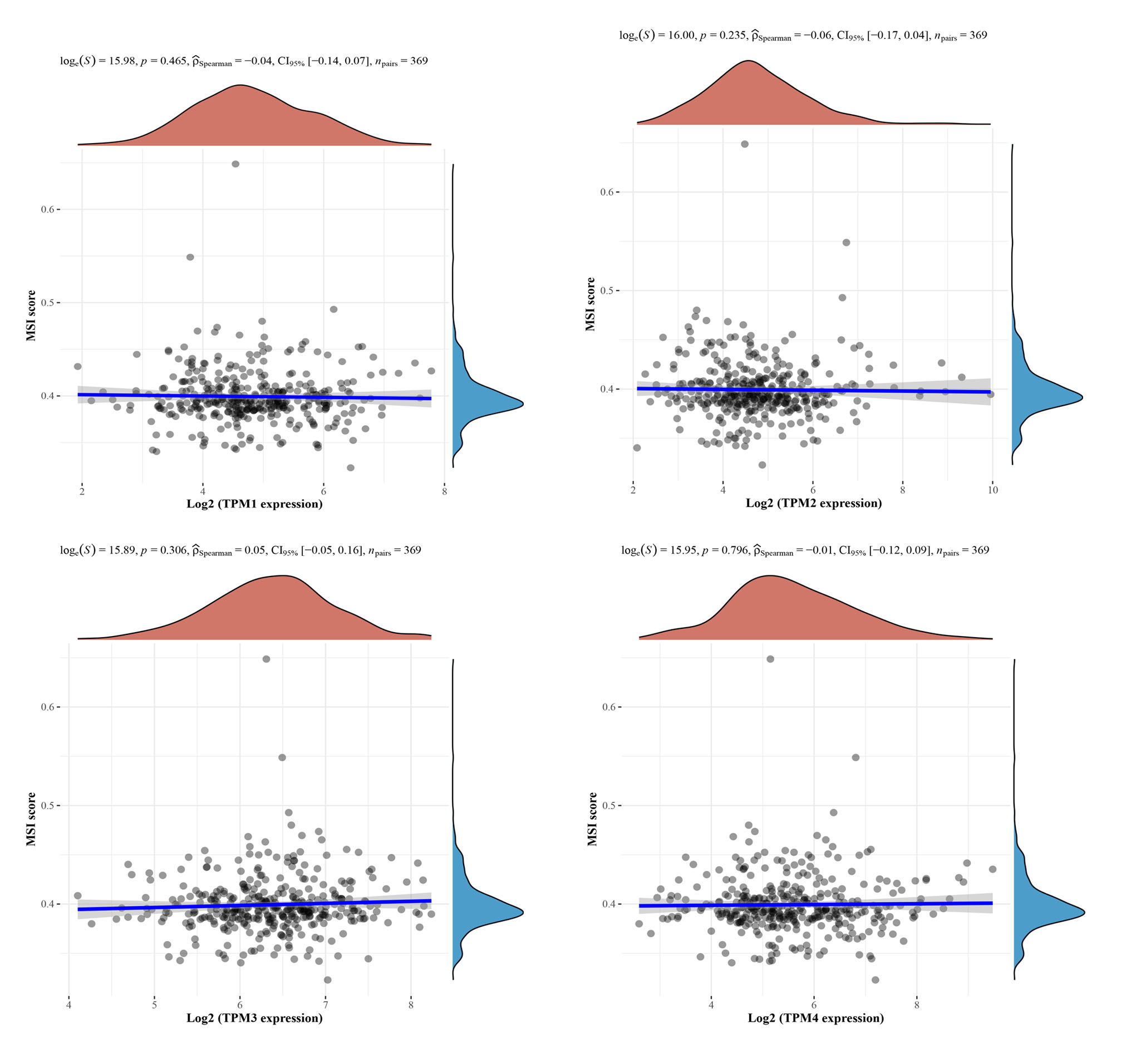

Supplement: Supplementary file 1 — Supplementary Material [file CAM4-11-433-s001.zip › cam44453-sup-0006-FigS6.jpg]

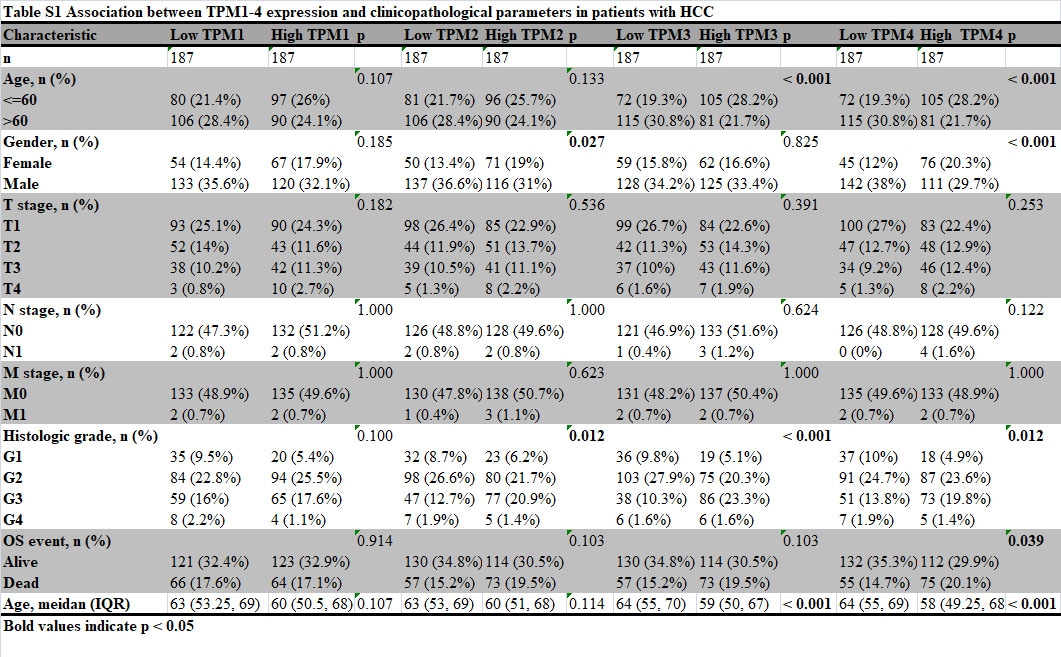

Supplement: Supplementary file 1 — Supplementary Material [file CAM4-11-433-s001.zip › cam44453-sup-0007-TableS1.jpg]

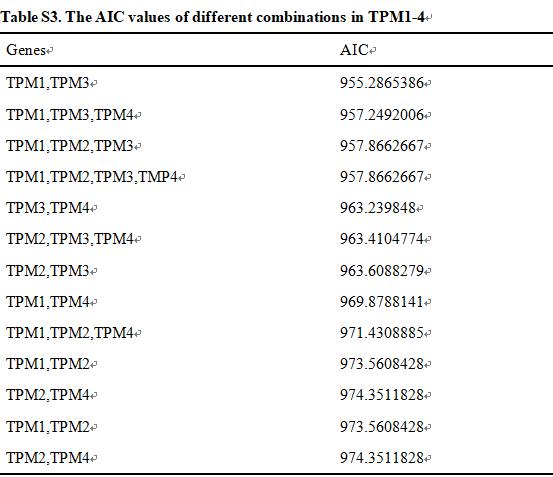

Supplement: Supplementary file 1 — Supplementary Material [file CAM4-11-433-s001.zip › cam44453-sup-0009-TableS3.jpg]
